# SMRT sequencing only *de novo* assembly of the sugar beet (*Beta vulgaris*) chloroplast genome

**DOI:** 10.1186/s12859-015-0726-6

**Published:** 2015-09-16

**Authors:** Kai Bernd Stadermann, Bernd Weisshaar, Daniela Holtgräwe

**Affiliations:** Chair of Genome Research, Faculty of Biology, Bielefeld University, Bielefeld, Germany; Bioinformatics Resource Facility, Centre for Biotechnology, Bielefeld University, Bielefeld, Germany

**Keywords:** Assembly, PacBio, SMRT sequencing, Sugar beet, Chloroplast, Sprai

## Abstract

**Background:**

Third generation sequencing methods, like SMRT (Single Molecule, Real-Time) sequencing developed by Pacific Biosciences, offer much longer read length in comparison to Next Generation Sequencing (NGS) methods. Hence, they are well suited for *de novo*- or re-sequencing projects. Sequences generated for these purposes will not only contain reads originating from the nuclear genome, but also a significant amount of reads originating from the organelles of the target organism. These reads are usually discarded but they can also be used for an assembly of organellar replicons. The long read length supports resolution of repetitive regions and repeats within the organelles genome which might be problematic when just using short read data. Additionally, SMRT sequencing is less influenced by GC rich areas and by long stretches of the same base.

**Results:**

We describe a workflow for a *de novo* assembly of the sugar beet (*Beta vulgaris ssp. vulgaris*) chloroplast genome sequence only based on data originating from a SMRT sequencing dataset targeted on its nuclear genome. We show that the data obtained from such an experiment are sufficient to create a high quality assembly with a higher reliability than assemblies derived from e.g. Illumina reads only. The chloroplast genome is especially challenging for *de novo* assembling as it contains two large inverted repeat (IR) regions. We also describe some limitations that still apply even though long reads are used for the assembly.

**Conclusions:**

SMRT sequencing reads extracted from a dataset created for nuclear genome (re)sequencing can be used to obtain a high quality *de novo* assembly of the chloroplast of the sequenced organism. Even with a relatively small overall coverage for the nuclear genome it is possible to collect more than enough reads to generate a high quality assembly that outperforms short read based assemblies. However, even with long reads it is not always possible to clarify the order of elements of a chloroplast genome sequence reliantly which we could demonstrate with Fosmid End Sequences (FES) generated with Sanger technology. Nevertheless, this limitation also applies to short read sequencing data but is reached in this case at a much earlier stage during finishing.

**Electronic supplementary material:**

The online version of this article (doi:10.1186/s12859-015-0726-6) contains supplementary material, which is available to authorized users.

## Background

During the last decade sequencing technologies took a large leap ahead. The so called Next Generation Sequencing (NGS) technologies [1, 2] offer a much higher amount of sequencing data in comparison to classical Sanger sequencing [3], while still delivering a read accuracy of over 99 %. Additionally, the cost per sequenced base is drastically reduced when using NGS technologies compared to Sanger sequencing [4]. However, NGS platforms suffer from a huge drawback: their read length is relatively short. Eukaryotic genomes often contain repetitive regions significantly longer than the maximum NGS read length, and due to the repeat structures also mate pair data cannot fully resolve these regions. This results in a lot of contigs that cannot be assigned to a distinct position during scaffolding. As a consequence, many of the genome sequences that were produced recently stay “unfinished” when compared to genome sequences generated by the BAC to BAC approach [5]. To overcome this issue third generation sequencing technologies seem to be well suited, as they deliver a much longer average read length. One representative of this technology has been developed by Pacific Biosciences and is called SMRT (Single Molecule, Real-Time) sequencing [6]. The newest PacBio RS II sequencer produces an average read length of 10 to 15 kb and a maximum read length of up to 64,500 bp [7]. The price one has to pay for these long reads is accuracy: the error rate of a SMRT read is about 11 % at the moment. However, in contrast to NGS technologies the errors produced by SMRT sequencing are not biased, they appear randomly. This means that these errors can easily be compensated by increased sequencing coverage [8]. The long reads have the potential to span even long repetitive regions and hence help to get these regions integrated into an assembly. Additionally, sequence motives that cannot be sequenced by NGS or even Sanger technology like longer single nucleotide runs or GC-rich regions can be read by SMRT sequencing [9]. This makes the technology a very good choice for creating *de novo* assemblies or for improving existing NGS assemblies given that sufficient coverage can be generated.

The DNA extracted from cells not only contains the nuclear genome but also DNA from organelles (e.g. the chloroplast in case of plants). Reads from this DNA make up a notable amount of the resulting raw reads. If the goal is the enhancement or assembly of the nuclear genome these reads are usually filtered out and discarded. However, they can also be used to assemble the organelles genome sequence. Sequences originating from organellar DNA usually make up a large percentage of the overall reads in relation to the organelles genome size due to higher copy numbers of organellar DNA. Consequently, the average coverage of organellar genomes is usually much higher than the average coverage of the nuclear genome [10, 11]. This provides the opportunity to perform a complete *de novo* assembly of the organellar genomes only based on SMRT sequencing data that have low coverage for the nuclear genome.

Here we present a SMRT sequencing only *de novo* assembly of the *Beta vulgaris ssp. vulgaris* chloroplast genome. As previously shown by Ferrarini et al. [12] SMRT data provide a great basis to create a high quality chloroplast genome assembly. We only used data originating from a low coverage resequencing project focussing on the nuclear genome. In contrast to other methods also based on SMRT sequencing [13–15], our method is based on data that is generated as a by-product during nuclear genome sequencing and no extra data needs to be generated. The workflow established for sugar beet is described in detail and is made available. Results were compared to the published sugar beet chloroplast assembly from Li et al. [16] which is based on the same genotype but only on Illumina sequencing. The chloroplast sequence is a good example to show the power of SMRT sequencing as it contains beside the Large Single Copy Region (LSCR) and the Small Single Copy Region (SSCR) two large inverted repeat (IR) regions that are hard to assemble using only short reads.

## Methods

### Plant material and DNA extraction

Genomic DNA for SMRT library construction was preparated from leaf material with a modified CTAB-DNA extraction method followed by QIAGEN Genomic-tip 100/G (Qiagen, Hilden, Germany) cleaning and filtering. The sugar beet DH plants of the sequenced genotype KWS2320 [17] were provided by KWS SAAT SE.

Plants were grown in the greenhouse under long day conditions on soil for 6 weeks. Reduction of starch content was performed by etiolation for 4 days prior to harvest. About 2.5 g young tissue was ground under liquid nitrogen and mixed with 20 ml prewarmed modified Carlson-buffer [18] containing 3 % CTAB, 3 μl/ml 2-ME and 0.2 mg RNAse. The homogenate was incubated at 74 °C for 30 min with inverting every 5 min. The DNA was than extracted with 1 Vol. chloroform:isoamylalcohol (24:1) and centrifuged with 17,000 rpm at RT. The aqueous phase was diluted with 1 Vol. H_2_O and adjusted to pH 7.0 prior to Genomic-tip 100/G purification and precipitation. The final pellet was resuspended in 500 μl sterile distilled water, the DNA concentration determined and the purity and integrity visualized on an agarose gel.

### Library construction and sequencing

The construction of the PacBio RS libraries with a targeted insert size of 8–12 kb and subsequent sequencing was outsourced to the sequencing provider GATC Biotech AG (Constance, Germany). The raw read data originates from two different sequencing runs. The first run was performed on a PacBio RS sequencer using C2 chemistry and XL polymerase on 10 SMRT-Cells. The data were delivered in 04/2013. The second run was performed on a PacBio RS II using P4 Polymerase and C2 chemistry on 15 SMRT-Cells. These data were delivered in 01/2014.

### Raw sequencing data

After extracting reads from the sequencing output files with the SMRT Analysis [19] toolkit using the “RS_Subreads” pipeline with standard settings, roughly 5.6 Gbp of data were available consisting of 1,741,381 subreads originating from the nuclear genome and organelle DNA. Additional file 1 shows the length distribution over all these subreads. The average subread length was 3213 bp with a N50 length of 4713 bp. Assuming a genome size of 731 Mbp for sugar beet this results in theoretically 7.6 fold coverage.

### Extraction of potential chloroplast reads

In order to find potential chloroplast reads in the pool of all available reads, the reads were mapped to the spinach (*Spinacia oleracea*) chloroplast genome sequence [20]. The program BLASR [21] was used for mapping using a minimal identity threshold for matched reads of 80 %. To speed up the mapping process the available read data were divided into 100 subsets and then mapped in parallel on a compute cluster. The mapping process resulted in one sam file per subset. In a next step samtools [22] was used to extract the IDs of the mapped reads from each sam file providing a first white list of reads most likely originating from the chloroplast genome. The subread IDs in this list were used to deduce polymerase read IDs that were required for further processing (HGAP Whitelisting Tutorial). As a polymerase read might contain more than one subread it is possible that after deducing some of the IDs appear multiple times. In a final step multiple polymerase read ID entries were consolidated into one single entry.

The white list was now used in combination with the SMRT Analysis toolkit to extract the subreads from the pool of all available read data. The advantage of having a white list is that all pipelines defined in SMRT Analysis can be used without the need to modify a lot of settings. It is sufficient to add the white list as a parameter to the filter module that is part of all pipelines. By doing so all later analysis steps are only performed with the reads defined in the white list.

### Assembly

For the assembly step the sprai assembly pipeline [23, 24] was used. All reads extracted in the preceding step were used as input. Sprai first performs an error correction of the SMRT reads. In a second step the error corrected reads are handed over to the Celera Assembler [25] for the final assembly step. After assembly the “check_circularity.pl” script that is also provided by the sprai authors was used to check if the resulting assembly has overlapping ends.

### Creation of coverage plots

In order to account for the chloroplast genome being circular, we created each coverage plot based on two mappings. The first mapping was performed against the normal cp_2320 sequence. Since reads that span the region where the assembly was linearized (to form a text string) will not map properly, we created a second mapping against the cp_2320 sequence that was linearized at the opposite position of the chloroplast genome consensus start and end point. From this second mapping, the coverage values corresponding to the first and the last 7000 bp of the original cp_2320 assembly were integrated into the final coverage plots.

### Validating, reordering and polishing the assembly

The assembly was validated using an existing library of Fosmid End Sequences (FES) [17, 26] created by Sanger sequencing from about 100.000 fosmid clones of the genotype KWS2320. To check the order of elements in the assembly a selected subset of 23 FES read pairs, selected for complete coverage at the clone level of the chloroplast genome, was used. The FES sequences were mapped to the chloroplast genome assemblies using bowtie2 [27] with standard settings.

As the newly created assembly starts at an arbitrarily defined position, it was reordered to align with the established start position of chloroplast genomes. This facilitates comparison of the new SMRT sequencing assembly with the earlier Illumina assembly [16]. In a final step the assembly was polished using the Quiver algorithm [28] and all reads white-listed during the first step. The corresponding pipeline was provided by the SMRT Analysis toolkit. After polishing the SMRT sequencing only assembly designated cp_2320 was finalized.

To preclude that nuclear DNA sequences did accidentally contribute parts of the assembly, we mapped all available SMRT reads against the final assembly and created a coverage plot. If a part of the assembly would be of nuclear origin only, a drastic drop in the coverage from about 2000 fold to about 10 fold should be visible. Such regions were not observed. However, it cannot be completely excluded that nuclear reads are included in the assembly process, because insertions of chloroplast DNA into the nuclear genome are known for plants [29]. If a nuclear read is very similar to a region on the spinach chloroplast genome sequence, it might survive the filtering step. These reads might even show small differences to the reads really originating from this position on the chloroplast. Nevertheless, these reads will represent a minority and during the polishing step they will be overruled by the vast majority of real chloroplast reads and hence they will not influence the final sequence of the assembly.

### Annotation of the assembly

The online service CpGAVAS [30] was used to create an annotation for the assembly cp_2320. As a reference for the annotation process, we used spinach. Aside from this change, we used the standard settings provided by CpGAVAS.

### Comparing the assemblies

The cp_2320 assembly was compared to the already available assembly by aligning the sequences against each other to identify differences between the two. For the alignment the EMBOSS stretcher application [31] was used. To check which assembly is correct we mapped all available FES generated by Sanger technology against both assemblies using bowtie2 [27], treating them as single end reads.

Changes in the annotation caused by differences of the underlying nucleotide sequence where examined using nucleotide and protein blast as well as blastx [32]. All blast variants where used through the NCBI web interface using standard settings. Nucleotide blast was run against the “Nucleotide collection (nt)” database; protein blast and blastx were run against the “Non-redundant protein sequences (nr)” database.

## Results and discussion

### Subread extraction

**Fig. 1:**
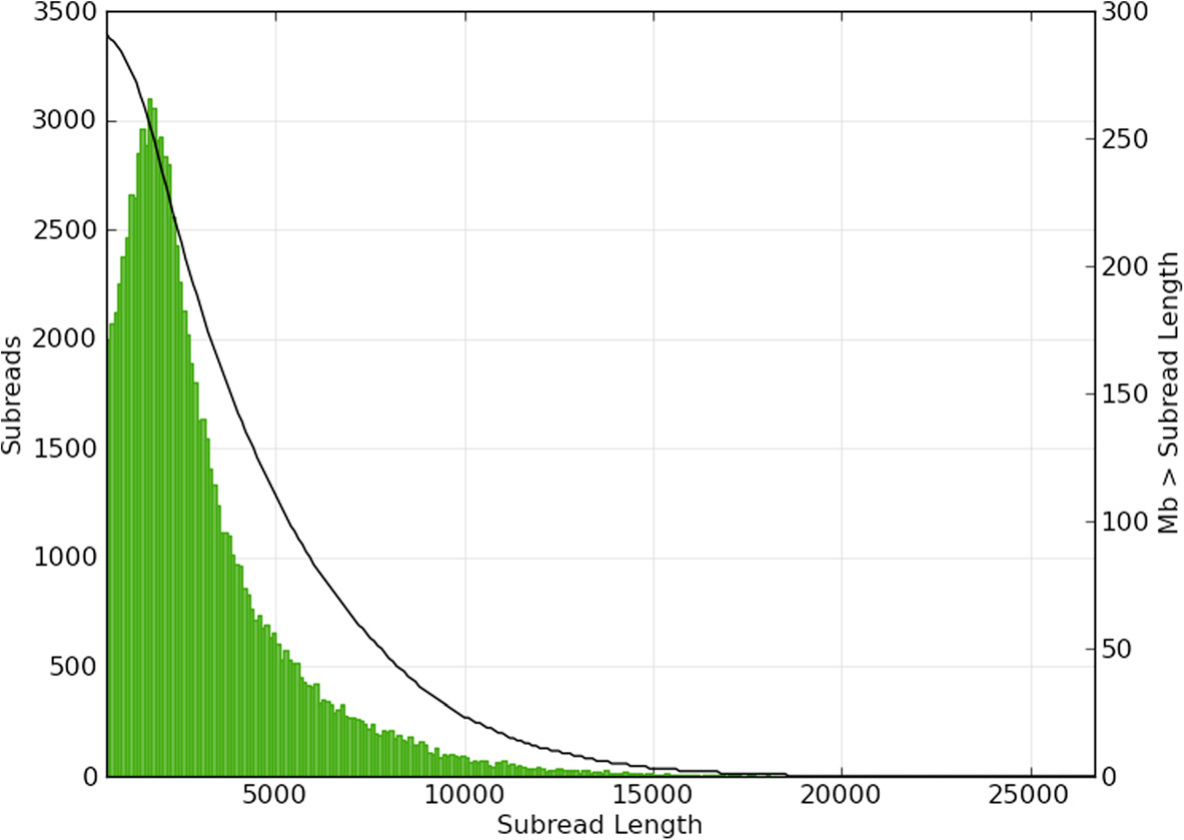
Length distribution of white list extracted subreads. The length distribution of all the subreads extracted from the complete dataset by using SMRT Analysis and the white list. Subread length is given in bp

The extraction of relevant chloroplast genome reads out of the whole SMRT sequencing data set yielded a total of 96,874 subreads containing 296,752,589 base calls (SRR1980665), representing 4.1 % of the total sequence yield. The distribution of subread length from chloroplast DNA is given in Fig. 1. Based on an expected chloroplast genome size of 150,000 bp this results in a total coverage of 1978 fold (see below).

### Result of the assembly process

The more than 96,000 subreads were used to create the sprai assembly. The first raw assembly showed overlapping ends, indicating that it was complete. The assembly was validated using a FES library [17, 26]. To check the order of elements in the assembly a selected subset of 23 FES read pairs (Additional file 2) spanning the complete chloroplast genome was used. After mapping the subset of read pairs to the assembly, the distance and the orientation of the corresponding read pairs were checked (Additional file 3). Most of the read pairs mapped in a consistent way, but read pairs with one partner read in the Large and the other one in the Small Single Copy Region showed inconsistent orientation and distance values (Table 1). These inconsistencies indicated that the Small Single Copy Region was included in the wrong orientation. The Large and Small Single Copy Region are connected by two IR regions, so their correct orientation could have only been determined by a read completely spanning one of the approximately 25,000 bp long repeats and reaching into each Single Copy Region. The data set only contains two reads, theoretically long enough but they do not span a repeat. Based on the information we gained from the FES alignment we inverted the Small Single Copy Region (Additional file 4), resulting in correct orientation of the former FES in question (Table 2). After quality polishing using Quiver the final cp_2320 assembly had a total length of 149,722 bp.

**Table 1:** Wrong FES orientation before reordering. Before reordering the FES pairs with one partner in the Small Singe Copy Region (SSCR) and the other one in the Large Single Copy Region (LSCR) they showed a wrong orientation. Position and length distances are given in bp

*Name*	*Position*	*Length*	*Orientation*	*Located on*	*Accession*
001-G11-CCfw	122,716	669	fw	SSCR	FI107918
001-G11-CCrv	27,487	778	fw	LSCR	FI107577
002-M15-CCfw	19,833	852	rv	SSCR	KG642400
002-M15-CCrv	60,551	780	rv	LSCR	KG642401
198-M21-ccrv	23,992	579	rv	SSCR	JY420463
198-M21-pIfw	53,203	617	rv	LSCR	JY420464

**Table 2:** Correct orientation of FES in question after reordering. After reordering the FES pairs with one partner in the Small Singe Copy Region (SSCR) and the other one in the Large Single Copy Region (LSCR) they now show the correct orientation towards each other. Position, length and distances are given in bp

*Name*	*Position*	*Length*	*Orientation*	*Distance*	*Located on*	*Accession*
001-G11-CCfw	70,072	669	fw	37,428	SSCR	FI107918
001-G11-CCrv	107,500	778	rv		LSCR	FI107577
002-M15-CCfw	115,079	852	fw	42,542	SSCR	KG642400
002-M15-CCrv	7899	780	rv		LSCR	KG642401
198-M21-ccrv	111,193	579	fw	39,080	SSCR	JY420463
198-M21-pIfw	551	617	rv		LSCR	JY420464

**Fig. 2:**
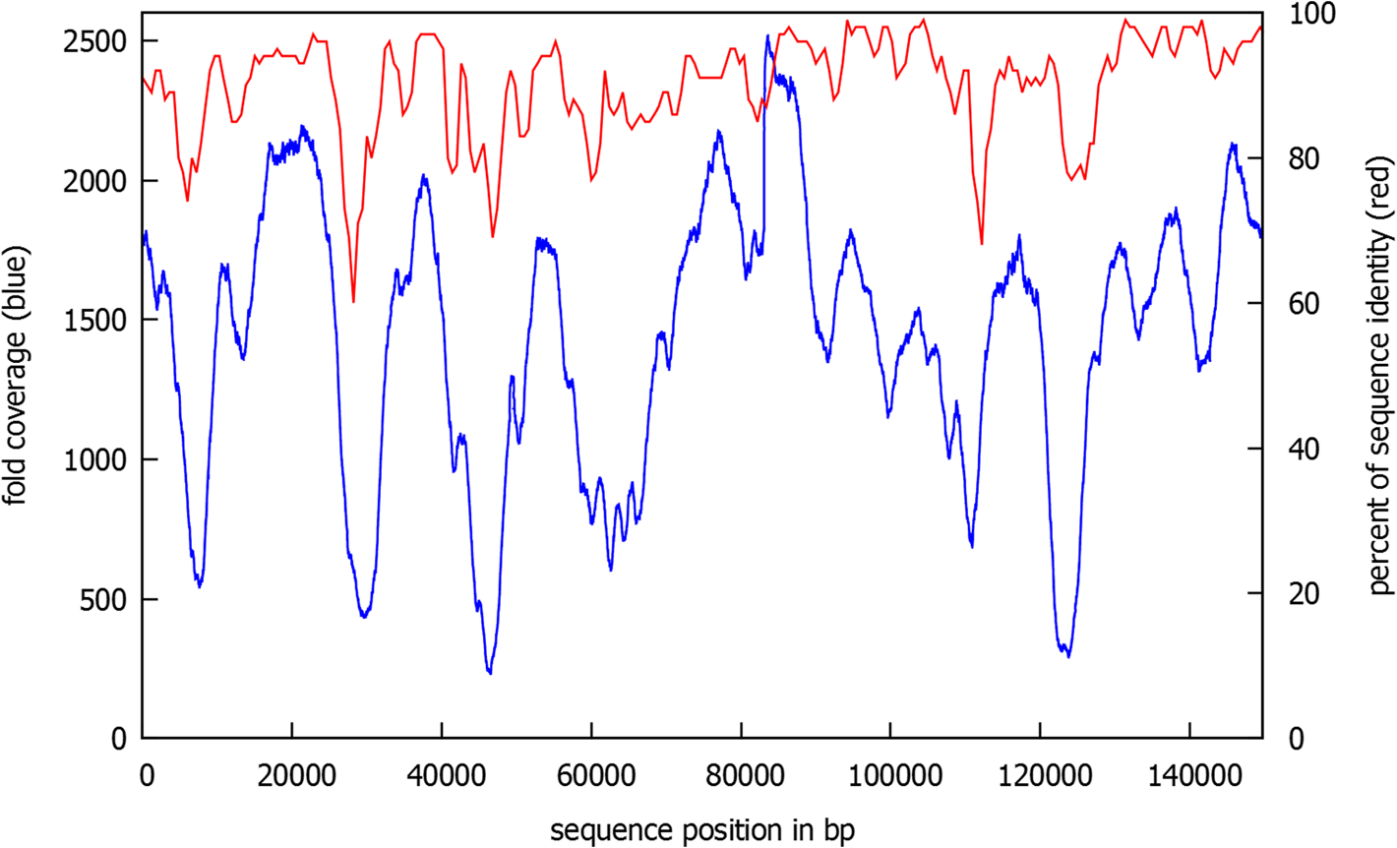
Correlation between extracted reads an sequence identity. The figure shows the correlation between the coverage of cp_2320 with extracted reads (blue) and the percentual sequence identity between cp_2320 and the spinach chloroplast (red)

Although the coverage of 1978 fold seems to be excessive at first, Fig. 2 shows that the reads were not distributed equally across the cp_2320 assembly (blue curve). To explain this distribution of the reads filtered on the basis the spinach chloroplast genome sequence, the sequence identity between the two assemblies was calculated (red curve). When comparing the two curves, a correlation between read coverage and sequence identity becomes obvious. Clearly, sequence regions with a higher degree of variation distance to the sugar beet chloroplast genome yield less reads than regions with high sequence similarity. Hence, a higher overall coverage is needed in comparison to a classical assembly. Nevertheless, even for less well covered regions the Pacific Biosciences recommendations of about 80 to 100 fold coverage for a high quality assembly [8] were easily reached.

**Fig. 3:**
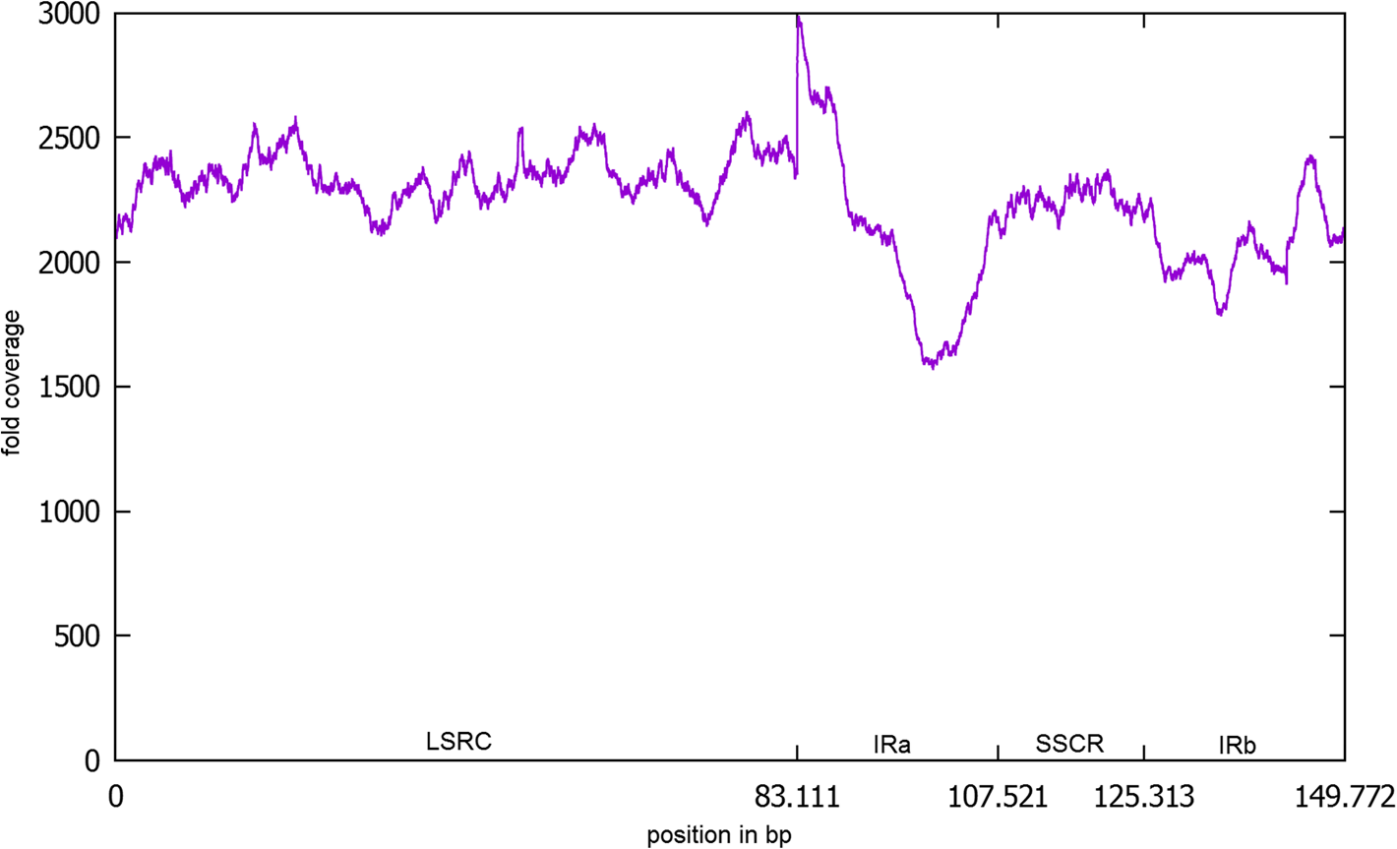
SMRT read coverage of cp_2320. The figure shows the SMRT read coverage of cp_2320. All available SMRT reads were mapped against cp_2320 using BLASR

To demonstrate that the true read coverage of cp_2320 in the original dataset is quite even, a mapping was performed with all SMRT reads (Fig. 3). This coverage was high and evenly distributed over the assembly, also indicating that no nuclear region with high sequence similarity to chloroplast sequences was accidently included. At the beginning of the first IR (position 83,111) a peak in the coverage was observed. We suspect that this peak is an artefact of the mapping process resulting from the repeat structure rather than a real increase of coverage.

### Comparison to the available sugar beet chloroplast assembly

**Fig. 4:**
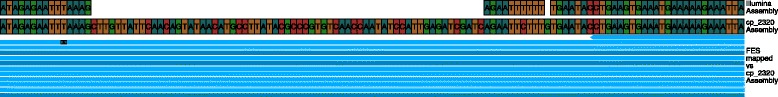
Difference between the Illumina assembly and the cp_2320 assembly. The figure shows one of the large differences between the Illumina and the cp_2320 assembly. The FES reads in blue have been mapped against the cp_2320 assembly. They match perfectly. When the reads are mapped against the Illumina assembly they show a large insertion. The Illumina assembly is aligned accordingly. The FESs shown are: JY285063, JY418627, JY362731, JY324111, JY312149, JY453558, JY373643, JY417666, JY300801, JY294801, JY285924

**Fig. 5:**
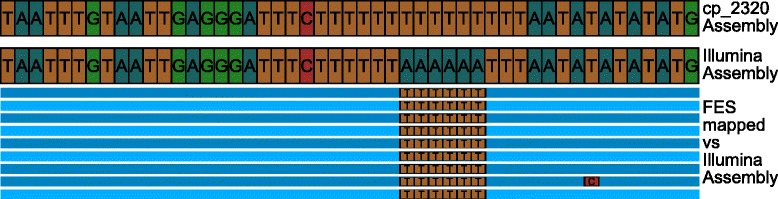
Wrong basecall in the Illumina assembly. The figure shows a mapping of the FES reads against the Illumina assembly. It is clear to see that the six “A” bases in the Illumina assembly are wrong as all the FES reads show six “T” at this position. The same position in the cp_2320 assembly is correct and shows no difference to the FESs. The FESs shown are: JY424769, JY376150, JY452900, JY393776, JY439089, JY393874, JY459728, JY449002, JY393875

The comparison of our cp_2320 assembly to the already available one revealed 23 differences between the assemblies: 14 small differences in a single position, 7 differences of 2 to 6 bp and two longer differences of 61 and 45 bp in length (see Figs. 4 and 5). Table 3 shows the complete list of differences between the two assemblies. Of the complete FES pool we could map 3279 reads consisting of 2,215,927 bp to the Illumina based reference and 3280 reads consisting of 2,216,647 bp to the newly created one. This results in a coverage with Sanger reads of about 15 fold for both assemblies. Each of the 23 differences was examined individually. For 22 differences the mapped Sanger reads supported the cp_2320 assembly. Only in one case the existing assembly was proven correct by the FES. A closer examination of this position reveals that it is highly supported by SMRT reads making a misassembly unlikely but still possible. The error corrected reads at this position revealed that there are indeed SMRT reads supporting both versions and hence this position remains ambiguous.

**Table 3:** Differences between the assemblies. This table lists all 23 differences between our assembly and the existing Illumina assembly. The validation results when taking the FES into account are also given

*Position cp_2320*	*Position KJ8018*	*Type*	*cp_2320 correct with respect to FES*	*Influenced annotation*	*Located in*
1355	1355	Insertion in cp_2320 (61 bp)	Yes	-	LSCR
1426	1361	Substitution (1 bp)	Yes	-	LSCR
1430	1365	Insertion in cp_2320 (1 bp)	Yes	-	LSCR
12,633	12,567	Insertion in cp_2320 (1 bp)	Yes	-	LSCR
29,255	29,188	Deletion in cp_2320 (5 bp)	Yes	-	LSCR
35,514	35,452	Deletion in cp_2320 (2 bp)	Yes	-	LSCR
45,756	45,696	Substitution (1 bp)	Yes	-	LSCR
45,773	45,713	Deletion in cp_2320 (1 bp)	Yes	-	LSCR
45,776	45,717	Deletion in cp_2320 (1 bp)	Yes	-	LSCR
45,780	45,722	Deletion in cp_2320 (1 bp)	Yes	-	LSCR
51,980	51,923	Deletion in cp_2320 (4 bp)	Yes	-	LSCR
56,686	56,633	Deletion in cp_2320 (1 bp)	Yes	accD	LSCR
60,118	60,066	Insertion in cp_2320 (2 bp)	Yes	cemA	LSCR
63,266	63,212	Substitution (1 bp)	Yes	psbL	LSCR
84,321	84,267	Insertion in cp_2320 (1 bp)	Yes	-	IR 1
96,607	96,552	Deletion in cp_2320 (6 bp)	Yes	-	IR 1
100,122	100,073	Deletion in cp_2320 (1 bp)	No	-	IR 1
111,817	111,769	Substitution 6 bp (1 bp)	Yes	-	SSCR
111,858	111,810	Insertion in cp_2320 (45 bp)	Yes	ccsA	SSCR
114,513	114,420	Substitution (1 bp)	Yes	ndhD	SSCR
117,806	117,713	Deletion in cp_2320 (1 bp)	Yes	ndhA^a^	SSCR
136,237	136,145	Deletion in cp_2320 (6 bp)	Yes	-	IR 2
148,514	148,428	Insertion in cp_2320 (1 bp)	Yes	-	IR 2

^a^Deletion occurs in intron and does hence not influence the coding region

### Annotation

**Fig. 6:**
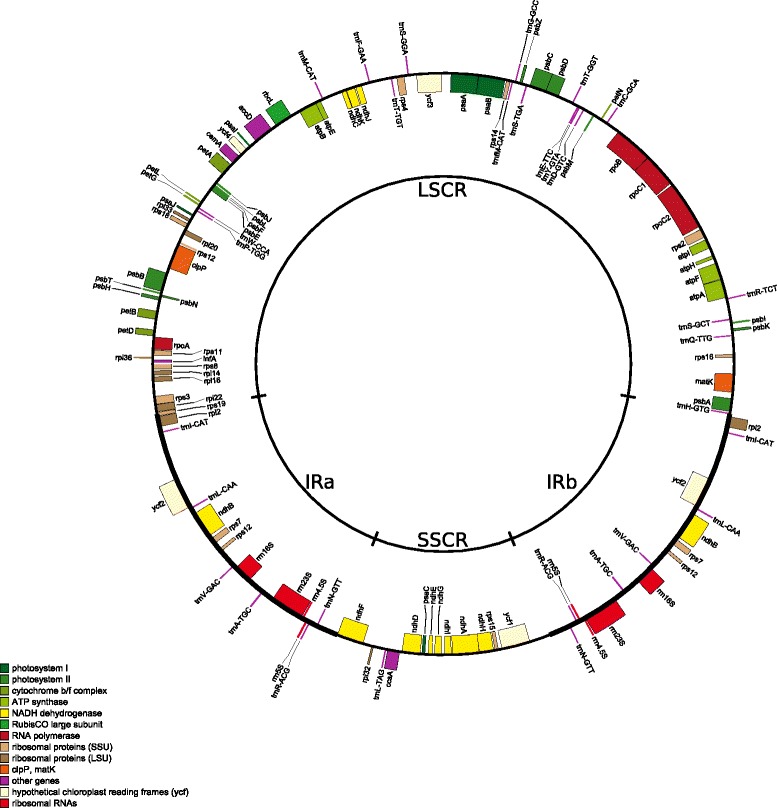
Annotation of the cp_2320 assembly. The figure gives an overview of the new annotation of the SMRT sequencing only *de novo* assembly cp_2320

Finally, the cp_2320 assembly was applied to a gene prediction and annotation approach; the result is shown in Fig. 6. A total of 114 individual genes were identified. Of these, 79 genes encode mRNA (i.e. proteins), 7 rRNA and 28 tRNA. Nine genes are located within the IR regions which encode 5 mRNA, 1 rRNA and 3 tRNA. In comparison to the Illumina assembly our annotation shows some differences caused by changes in the underlying sequence.

The genes *psbL* and *ndhD* show a different start codon. In the Illumina assembly they start with the classical “ATG” codon, whereas in cp_2320 they start with the codon “ACG”. For *psbL* “ACG” has been reported as an initiator codon in tobacco (*Nicotiana tabacum*) and spinach. By mRNA editing from C to U the “AUG” initiator is formed [33]. This effect is also known for the *ndhD* gene [34].

The gene *accD* is influenced by a deletion in the cp_2320 assembly causing a change in the reading frame. However, the accD domain is not influenced by this change and high scoring blast results exist for our gene annotation.

The gene *cemA* is influenced by a two base insertion in the cp_2320 assembly at the beginning of the predicted gene causing our annotation to start one amino acid later with a stretch of 3 lysines followed by a methionine. In spinach this methionine is the start amino acid of the cemA protein and it is hence likely that the real translation start is located at this position.

The gene *ccsA* shows the biggest change, as it is located at the side of the 45 bp insertion. The insertion causes one amino acid change followed by an insertion of 15 new amino acids. The new amino acid sequence can be found exactly in spinach and in almost all other blast hits.

## Conclusions

SMRT sequencing reads extracted from a pool of reads created for nuclear genome (re)sequencing have been used to obtain a high quality consensus sequence of the chloroplast from sugar beet. Even with a relatively small overall coverage of total genomic DNA it was possible to collect more than enough reads to generate a high quality *de novo* assembly of the chloroplast genome. Comparison to a published assembly from the same genotype generated with Illumina data indicated that short read based genome sequences may contain errors. These were mostly caused by specific features of the sequences, like longer stretches of the same base (Fig. 5). High coverage SMRT sequencing turned out to be almost error-free when accepting Sanger data as “golden standard”.

If the genome in question contains large repetitive elements also SMRT sequencing reaches its limits. When the repeat is too long to be spanned by SMRT reads these elements cannot be integrated in assemblies in a reliable way. In the case presented here, this problem was overcome with FES long-read paired end data offering about 40 kbp long “jumps” generated by Sanger. However, when using newer versions of the Pacific Biosciences chemistry and size-selected libraries, even longer reads will become more common and repetitive elements might be resolved automatically during the assembly process.

## Availability of supporting data

The extracted SMRT sequencing raw reads are available in the “Short Read Archive” under accession ID SRR1980665. The sequence and annotation of the cp_2320 chloroplast assembly have been submitted to GenBank and are available under accession ID KR230391. Additional data sets supporting the results of this article are included within the article (and its additional files).

## Additional files

Additional file 1:
**Distribution of read length of all subreads.** The distribution of read length of all the subreads contained in the complete SMRT sequencing dataset. (PDF 134 kb) Additional file 2:
**FES internal names to GenBank Accession IDs.** This file provides a translation from our internally used FES identifiers to the publically available GenBank Accession IDs. (PDF 28 kb) Additional file 3:
**FES orientation before reordering.** This file contains the orientation and position of all 23 FES pairs used to validate the assembly. The three pairs showing a wrong orientation towards each other are marked in red. (PDF 25 kb) Additional file 4:
**FES orientation after reordering.** This file contains the orientation, position and distance of all 23 FES pairs towards each other after reordering the assembly. All pairs now show correct distance and orientation towards each other. However, there are some cases where one partner is located in a repeat region. In this case of course only one distance is correct. (PDF 25 kb)

## Abbreviations

SMRT: Single molecule, real time
NGS: Next generation sequencing
IR: Inverted repeat
FES: Fosmid end sequence
LSCR: Large single copy region
SSCR: Small single copy region
