# Supplementary material for: SMRT sequencing only de novo assembly of the sugar beet (Beta vulgaris) chloroplast genome
Source: BMC Bioinformatics. 2015 Sep 16;16(1):295. doi: 10.1186/s12859-015-0726-6 (PMC4573686; doi:10.1186/s12859-015-0726-6)

Additional file 1 - Distribution of read length of all subreads.

The distribution off read length of all the subreads contained in the complete SMRT sequencing dataset. The subread length is given in bp.

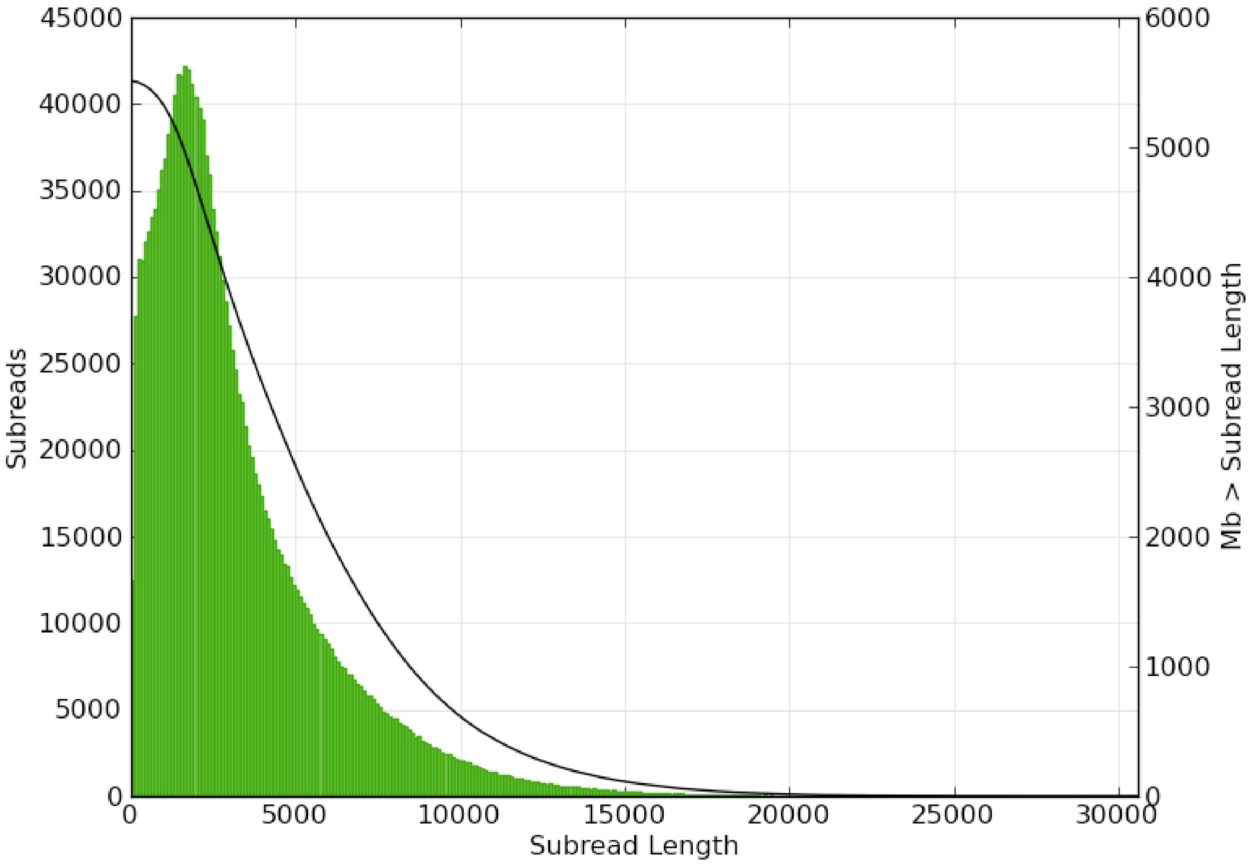

Supplement: Additional file 1: — Distribution of read length of all subreads. The distribution of read length of all the subreads contained in the complete SMRT sequencing dataset. (PDF 134 kb) [file 12859_2015_726_MOESM1_ESM.pdf]
