# Supplementary material for: SMRT sequencing only de novo assembly of the sugar beet (Beta vulgaris) chloroplast genome
Source: BMC Bioinformatics. 2015 Sep 16;16(1):295. doi: 10.1186/s12859-015-0726-6 (PMC4573686; doi:10.1186/s12859-015-0726-6)
Supplement: Additional file 2: — FES internal names to GenBank Accession IDs. This file provides a translation from our internally used FES identifiers to the publically available GenBank Accession IDs. (PDF 28 kb) [file 12859_2015_726_MOESM2_ESM.pdf]

Internal Name	GenBank Accession ID
001-G11-CCfw	FI107918
001-G11-CCrv	FI107577
002-M15-ccfw	KG642400
002-M15-ccrv	KG642401
011-K06-ccfw	JY290096
011-K06-ccrv	JY289812
019-O16-ccfw	JY297338
019-O16-ccrv	JY297339
022-D24-ccfw	JY315368
022-D24-ccrv	JY315369
032-D03-ccfw	JY280091
032-D03-ccrv	JY280092
039-L19-ccfw	JY299992
039-L19-ccrv	JY299993
059-D14-ccfw	JY323805
059-D14-ccrv	JY323806
080-J05-ccfw	JY333746
080-J05-ccrv	JY333747
092-L03-ccfw	JY340936
092-L03-ccrv	JY340937
110-O21-ccfw	JY364777
110-O21-ccrv	JY364778
128-L03-ccfw	JY374402
128-L03-ccrv	JY374403
151-P14-ccfw	JY404985
151-P14-ccrv	JY404986
172-B21-ccfw	JY390546
172-B21-ccrv	JY390547
179-N18-ccfw	JY410054
179-N18-ccrv	JY410055
198-M21-ccrv	JY420463
198-M21-pIfw	JY420464
227-J20-ccrv	JY412561
227-J20-pIfw	JY412562
238-F10-ccrv	JY453605
238-F10-pIfw	JY453606
240-J19-ccrv	JY439939
240-J19-pIfw	JY439940
275-H06-ccrv	JY468069
275-H06-pIfw	JY468070
278-G12Q-ccrv	JY463405
278-G12Q-pIfw	JY463406
279-D09-ccrv	JY275676
279-D09-pIfw	JY275677
279-I18-ccrv	JY276117
279-I18-pIfw	JY276118
