# Supplementary material for: SMRT sequencing only de novo assembly of the sugar beet (Beta vulgaris) chloroplast genome
Source: BMC Bioinformatics. 2015 Sep 16;16(1):295. doi: 10.1186/s12859-015-0726-6 (PMC4573686; doi:10.1186/s12859-015-0726-6)
Supplement: Additional file 3: — FES orientation before reordering. This file contains the orientation and position of all 23 FES pairs used to validate the assembly. The three pairs showing a wrong orientation towards each other are marked in red. (PDF 25 kb) [file 12859_2015_726_MOESM3_ESM.pdf]

Name	Location	Length	Orientation	Comment
001-G11-CCfw	122716	669	fw	located on sscr
001-G11-CCrv	27487	778	fw	located on lscr
002-M15-CCfw	19833	852	rv	located in sscr
002-M15-CCrv	60551	780	rv	located on lscr
011-K06-ccfw	110211	612	fw	
011-K06-ccrv	39746	634	fw	located in repeat
011-K06-ccrv	148028	634	rv	located in repeat
019-O16-ccfw	111934	724	fw	
019-O16-ccrv	39301	767	fw	located in repeat
019-O16-ccrv	148340	767	rv	located in repeat
022-D24-ccfw	124857	708	rv	
022-D24-ccrv	85672	481	fw	
032-D03-ccfw	105376	804	rv	
032-D03-ccrv	67003	853	fw	
039-L19-ccfw	61878	831	fw	
039-L19-ccrv	99926	832	rv	
059-D14-ccfw	7562	722	rv	located in repeat
059-D14-ccfw	30412	722	fw	located in repeat
059-D14-ccrv	120436	855	fw	
080-J05-ccfw	43145	762	fw	located in repeat
080-J05-ccfw	144501	762	rv	located in repeat
080-J05-ccrv	106333	642	fw	
092-L03-ccfw	122162	609	fw	
092-L03-ccrv	8757	707	rv	located in repeat
092-L03-ccrv	29231	707	fw	located in repeat
110-O21-ccfw	123347	642	fw	
110-O21-ccrv	9185	783	rv	located in repeat
110-O21-ccrv	28727	783	fw	located in repeat
128-L03-ccfw	7421	520	rv	located in repeat
128-L03-ccfw	30755	520	fw	located in repeat
128-L03-ccrv	118787	600	fw	
151-P14-ccfw	115481	695	fw	
151-P14-ccrv	1909	687	rv	located in repeat
151-P14-ccrv	36101	687	fw	located in repeat
172-B21-ccfw	118303	743	rv	
172-B21-ccrv	78542	695	fw	
179-N18-ccfw	130518	339	rv	
179-N18-ccrv	93410	323	fw	
198-M21-ccrv	23992	579	rv	located in sscr
198-M21-plfw	53203	617	rv	located on lscr
227-J20-ccrv	2997	760	rv	located in repeat
227-J20-ccrv	34939	760	fw	located in repeat
227-J20-plfw	117302	715	fw	
238-F10-ccrv	111307	773	fw	
238-F10-plfw	39988	840	fw	located in repeat
238-F10-plfw	147579	840	rv	located in repeat
240-J19-ccrv	133716	458	fw	
240-J19-plfw	18588	285	fw	
275-H06-ccrv	8833	727	rv	located in repeat

275-H06-ccrv	29135	727	fw	located in repeat
275-H06-plfw	67042	746	rv	
278-G12Q-ccrv	116127	602	fw	
278-G12Q-plfw	4448	603	rv	located in repeat
278-G12Q-plfw	33644	603	fw	located in repeat
279-D09-ccrv	81166	632	rv	
279-D09-plfw	45657	630	fw	located in repeat
279-D09-plfw	142121	630	rv	located in repeat
279-I18-ccrv	4119	734	fw	located in repeat
279-I18-ccrv	33842	734	rv	located in repeat
279-I18-plfw	40754	836	rv	located in repeat
279-I18-plfw	146822	836	fw	located in repeat
