# Supplementary material for: SMRT sequencing only de novo assembly of the sugar beet (Beta vulgaris) chloroplast genome
Source: BMC Bioinformatics. 2015 Sep 16;16(1):295. doi: 10.1186/s12859-015-0726-6 (PMC4573686; doi:10.1186/s12859-015-0726-6)
Supplement: Additional file 4: — FES orientation after reordering. This file contains the orientation, position and distance of all 23 FES pairs towards each other after reordering the assembly. All pairs now show correct distance and orientation towards each other. However, there are some cases where one partner is located in a repeat region. In this case of course only one distance is correct. (PDF 25 kb) [file 12859_2015_726_MOESM4_ESM.pdf]

Name	Location	Length	Orientation	Distance
001-G11-CCfw	70072	669	fw	37428
001-G11-CCrv	107500	778	rv	
002-M15-CCfw	115079	852	fw	42542
002-M15-CCrv	7899	780	rv	
011-K06-ccfw	57564	612	fw	37820
011-K06-ccrv	95384	634	rv	
011-K06-ccrv	136816	634	fw	108290
019-O16-ccfw	59287	724	fw	36409
019-O16-ccrv	95696	767	rv	
019-O16-ccrv	136371	767	fw	109047
022-D24-ccfw	72213	708	rv	39191
022-D24-ccrv	33022	481	fw	
032-D03-ccfw	52729	804	rv	38376
032-D03-ccrv	14353	853	fw	
039-L19-ccfw	9226	831	fw	38053
039-L19-ccrv	47279	832	rv	
059-D14-ccfw	104630	722	rv	
059-D14-ccfw	127481	722	fw	126871
059-D14-ccrv	67792	855	fw	36838
080-J05-ccfw	91857	762	rv	
080-J05-ccfw	140215	762	fw	101364
080-J05-ccrv	53686	642	fw	38171
092-L03-ccfw	69518	609	fw	36308
092-L03-ccrv	105826	707	rv	
092-L03-ccrv	126300	707	fw	129248
110-O21-ccfw	70703	642	fw	35551
110-O21-ccrv	106254	783	rv	
110-O21-ccrv	125796	783	fw	130230
128-L03-ccfw	104489	520	rv	126387
128-L03-ccfw	127824	520	fw	
128-L03-ccrv	66142	600	fw	38347
151-P14-ccfw	62835	695	fw	36141
151-P14-ccrv	98976	687	rv	
151-P14-ccrv	133171	687	fw	115527
172-B21-ccfw	65658	743	rv	39766
172-B21-ccrv	25892	695	fw	
179-N18-ccfw	77874	339	rv	37112
179-N18-ccrv	40762	323	fw	
198-M21-ccrv	111193	579	fw	39080
198-M21-plfw	551	617	rv	
227-J20-ccrv	100065	760	rv	117828
227-J20-ccrv	132009	760	fw	
227-J20-plfw	64656	715	fw	35409
238-F10-ccrv	58660	773	fw	36275
238-F10-plfw	94935	840	rv	
238-F10-plfw	137058	840	fw	107599
240-J19-ccrv	81072	458	fw	35819
240-J19-plfw	116891	285	rv	
275-H06-ccrv	105902	727	rv	129420

275-H06-ccrv	126204	727	fw	
275-H06-plfw	14392	746	rv	37910
278-G12Q-ccrv	63481	602	fw	38035
278-G12Q-plfw	101516	603	rv	
278-G12Q-plfw	130714	603	fw	120524
279-D09-ccrv	28516	632	rv	35511
279-D09-plfw	89477	630	rv	96472
279-D09-plfw	142727	630	fw	
279-I18-ccrv	130912	734	rv	36734
279-I18-plfw	94178	836	fw	
279-I18-plfw	137824	836	rv	36637
279-I18-ccrv	101187	734	fw	
